# The Prevalence of Dental Anxiety Associated with Pain among Chinese Adult Patients in Guangzhou

**DOI:** 10.1155/2021/7992580

**Published:** 2021-06-12

**Authors:** Jiali Yu, Rui Jiang, Er-Min Nie, Chun-Yuan Zhang, Xiang Li

**Affiliations:** First Affiliated Hospital, Sun Yat-Sen University, 58 Zhongshan 2nd Road, Guangzhou 510080, China

## Abstract

**Background:**

Anxious people appear to exaggerate the severity of aversive experiences such as anxiety and pain. Anxiety towards dental procedures is a common difficulty that may be experienced by dental patients all over the world. The goal of the study is to find out the prevalence of dental anxiety and its associated factors in Chinese adult patients.

**Methods:**

A cross-sectional study was conducted on 183 dental adult patients whose age ranged from 18 to 70 years. Demographic details, first and most recent dental visits with experience, the MDAS, and the Visual Analogue Scale for Anxiety (VAS-A) were obtained. Data were analyzed by frequency analysis, chi-square test, and Spearman correlation test.

**Results:**

Most of the respondents were female (68.9%) and 30–45 years age group. The mean total score for dental anxiety on the MDAS was 13.63 (3.1). 80.3% of participants suffered from moderate or high dental anxiety. Age must show a strong association with dental anxiety among the participants (*p*=0.011). The first dental visit experience, the frequency of the dental visit, most recent dental experience, length of time since the most recent dental visit, and postponement of the dental visit are strongly associated with the MDAS score (*p*=0.001).

**Conclusions:**

The MDAS score exhibits that Chinese adult patients have significant dental anxiety and phobia. Identifying patients with dental anxiety as soon as possible is essential to providing better dental care.

## 1. Introduction

Dental anxiety is a distress and unease situation that occurred in patients against the dental treatment procedure [1]. Despite modern and technical advancements in dentistry, existing research shows that dental anxiety remains a concern. Amid modern dental innovation and technical advancements, dental anxiety continues to be a major issue affecting both children and adults. Several studies have found a “dynamic vicious cycle” linking dental anxiety to poor oral health [2, 3]. Dental anxiety comprises a large proportion of people of all ages and social backgrounds, which often leads to poor oral health due to total avoidance of dental care, inconsistent dental attendance, or poor engagement [4]. Patients who suffer from dental anxiety are more likely to postpone or neglect dental treatment, resulting in the deterioration of their oral health. The progression of untreated oral infections, combined with feelings of remorse, humiliation, or worthlessness, contributes to an increase in dental anxiety, and the vicious cycle continues [3]. According to a recent study, 83.1 percent of Chinese adult patients had moderate to severe dental anxiety, and 16.2 percent met the requirements for specific dental phobia [5]. Patients suffering from dental anxiety are a significant source of stress that can jeopardize the dentist's clinical efficiency. As a result, patients with dental anxiety must be identified before treatment initiation. It will assist dental care professionals in breaking the cycle and providing successful treatment [6].

The Modified Dental Anxiety Scale (MDAS) is a reliable and factual tool used in clinical settings to assess dental anxiety [7]. MDAS is an extension of Corah's Dental Anxiety Scale (CDAS), a 4-item tool that asks patients to quantify their anxiety levels in four different dental circumstances. Humphris et al. suggested that MDAS improves on the original CDAS by including a question item about receiving local anesthetic injections and requesting the potential answers to each question on a Likert scale ranging from “not anxious” to “extremely anxious.” [7] The MDAS has the benefit of being a cost-effective tool for population-based research due to its simplicity [8]. Dental anxiety and fear have a detrimental effect on oral and dental health these days. It also causes complications and increases in costs. Based on the existing literature findings, we aimed to investigate the prevalence of dental anxiety and the factors that could induce dental anxiety in Chinese adult patients who visited our dental clinic at Guangzhou. We assume that the findings can also be used to minimize dental anxiety.

## 2. Methods

183 Chinese adult patients in First Affiliated Hospital, Sun Yat-Sen University, were included in this study according to inclusion criteria. Each participant received written informed consent and signed before participating to this study.

Each participant was given a set of questionnaires adopted from Dou et al. including three sections in this cross-sectional study [5]. In the first section, the question was regarding sociodemographics, oral health practice, and dental visits. Assessment of dental anxiety was done in the second section of the questionnaire. A Chinese version of the Modified Dental Anxiety Scale (MDAS) was adopted from Dou et al. [5] and divided into a 5-item measure to assess fear of dental procedures, including before going for treatment, waiting for treatment, drilling, cleaning, and local anesthetic injections. Ratings of 1–5 indicated “not anxious,” “slightly anxious,” “fairly anxious,” “very anxious,” and “extremely anxious” correspondingly. Dental fear can be described when the total MDAS score was 19 or above [4]. The researchers were trained and calibrated before this study, and the reliability of the intraexaminer was evaluated using the kappa test. A kappa value of 0.780 was achieved. Visual Analogue Scale (VAS) was used in the third section to assess the pain level for dental experiences.

All statistical analyses were performed by IBM SPSS v.26. The mean total MDAS score was calculated for all the categorized variables. Descriptive and frequency analyses were performed on all variables. A chi-square test was performed to compare the mean MDAS score between categories in the same variable. Tukey's post hoc test was performed to control for multiple comparisons. Spearman rank correlation was done to assess the strength of association between MDAS and variables.

## 3. Results

Table 1 shows the characteristics of the study participants. Among the 183 respondents, 31.1% were males and 68.9% were females. Most of the patients were 30–45 years age group (36.1%). The mean total score for dental anxiety on the MDAS was 13.63 (3.1). Based on the MDAS score, 19.7% of the subjects were identified to be less anxious (5–9 total score), 69.9% were moderately anxious (10–18 total score), and 10.4% were seriously anxious (≥19 total score). Most of the participants had an average level of self-perceived oral health (55.2%). Most of them went to the dentist for the first time when they were 12–18 years old (44.8%) with not bad (42.1%) to bad experiences (27.3%). Among the respondents, 75 had reported bad experiences in their most recent dental visit. More than half of the participants (60.1%) had reported that they postponed their dental visit.

Table 2 shows the participant responses for each MDAS item. Before going to treatment, 39.9% did not feel anxious, while 30.1% were anxious. Nearly half of the participants (49.2%) reported that they do not feel anxious during waiting for treatment following others who felt fairly anxious (40.4%). More than half of the participants reported that they feel very anxious during drilling (60.1%). Half of the respondents (50.3%) reported that they were extremely anxious about local anesthetic injections following very anxious (19.7%).

Table 3 shows the chi-square result of the MDAS score and other variable associations. Age must show a strong association with dental anxiety among the participants (*p*=0.011). The first dental visit is also associated with developing dental anxiety (*p*=0.012). Having a bad experience during the first dental visit was a contributing factor for anxiety. Frequency of the dental visit, most recent dental experience, length of time since the most recent dental visit, and postponement of the dental visit are strongly associated with the MDAS score (*p*=0.001).

Table 4 shows the correlation between the MDAS score and other variables. No correlation was found between the MDAS and variables including age, gender, educational background, employment status, and self-perceived oral health status. Negative dental experience during treatment demonstrated a strong relation with dental anxiety. Participants who had experiences of the less frequent dental visit were more anxious compared to those with more frequent visits (*p*=0.001). Pain at the most recent dental visit or before the present dental visit was an important factor correlating with dental anxiety among participants.

## 4. Discussion

“Dental anxiety” and “dental phobia” create a major problem for both patients and dentists. These two words are often used synonymously; however, there are key differences between them. Dental anxiety is characterized as a specific patient response to dental procedure-related stress when the trigger is unclear, ambiguous, or not present at the time [9]. Dental phobia is described as intense and constant anxiety in a dental setting, which causes the person to avoid going to the dentist at all costs unless a physical issue becomes overwhelming [10]. It is recommended that dental practitioners evaluate dental anxiety and dental phobia during clinical assessments using a valid and reliable scale that can accurately measure the subjective experience of dental anxiety and phobia [10].

The results of the present study indicate an increase in dental anxiety among patients with pulpal pain as compared to previous studies [10, 11]. Our findings have shown that young patients were more likely to experience dental anxiety and phobia than older patients. Furthermore, past negative experiences in childhood and adolescence, periodontal problem perception, and feeling the gag reflex during dental care were risk factors for dental anxiety and dental phobia. In our study, 83.3% of the subjects reported moderate to extreme dental anxiety, which is higher than the results of previous studies. Another Chinese study by Duo et al. found that 83.1% of people had moderate to severe dental anxiety [5]. According to Esmaeili et al.'s [12] Brazilian report, more than half of the patients (60.4%) were moderately or highly anxious. Different studies also show different prevalence rates of dental anxiety. The dental phobia criteria (MDAS score >19) were met by 10.2% of participants in our study, which was comparatively higher than many other previous studies [13, 14]. The findings' incompatibility can be due to variations in the cultural background and the dental features of the patients in the above study.

The most important predictors of dental anxiety and phobia were age and a past negative experience in a dental clinic. Many studies have shown that females are more afraid of dental treatment than males [14, 15]. Other related research, however, found no gender differences in dental anxiety [16, 17]. In our study, no significant differences in dental anxiety were found between men and women, supporting the results of previous studies conducted in Tanzania and Nepal [16, 17]. The relationship between age and the level of dental anxiety is still uncertain in the literature, and researchers have suggested conflicting findings. In the current study, the effect of age on the MDAS score was significant, with older patients scoring significantly lower than younger patients. This finding is consistent with the findings of many other related studies [9, 14, 16, 18]. Patients seeking pain relief were always fearful of the pain they would experience during the assessment and treatment procedure. Invasive procedures include local anesthetic injection, drilling the tooth, and removing the dental pulp [19]. Pain is a significant factor in dental anxiety. According to the literature, the primary cause of dental phobia is anxiety regarding pain during dental treatment [20]. The effect of a previous bad experience in a dental clinic on the MDAS score was also significant, with patients who had a previous bad experience in their first dental visit or most recent dental visit scoring substantially higher than patients who had no such experience. This result is completely consistent with the results of other studies [21–23]. A significant difference was found in terms of the frequency of visits to a dentist and length of time since the recent dental visit. Other researchers have reported similar results, claiming that patients who visit the dentist daily are less likely to exhibit dental anxiety [15].

The current study's findings must be interpreted considering many methodological limitations. Because of the convenience sampling technique used to choose patients and the lack of randomization, there is a risk of selection bias. Within the limitation of this research, it should be noted that this is a cross-sectional study, which makes it impossible to analyse causal relationships. Dental practitioners play an important role in the treatment and prevention of dental anxiety. It has been reported that people who suffer from dental anxiety can have a detrimental impact on their tooth examination and oral health [11]. We can avoid problems by asking the factors that can cause distress in patients before dental examinations, and we can estimate the risk to people's oral health. Future research is recommended to allow for further investigation and confirmation of the study's findings. A prospective study will support both dentists and patients, with the potential to address the drawbacks of a cross-sectional study.

## 5. Conclusion

Dental anxiety is common among Chinese adult patients. The results of this study support the hypothesis that people who are predisposed to fearfully react to pain are more likely to become stuck in a vicious cycle of anxiety, fear of pain, and avoidance of dental care. Younger age people, first time dental treatment seekers, and having previous bad experience are the main risk factors for dental anxiety found in our study. Addressing these aspects can enhance the efficiency of strategies for reducing anxiety and phobia in adult dental patients.

## Figures and Tables

**Table 1:** Characteristics of the study participants.

Variables		Frequency	Percentage (%)
Gender	Male	57	31.1
	Female	126	68.9

Age (years)	18–30	57	31.1
	30–45	66	36.1
	46–60	48	26.2
	60–70+	12	6.6

Educational level	Uneducated	36	19.7
	High school	67	36.6
	Degree/diploma	59	32.2
	Postgraduation	21	11.5

Employment	Employed (full time)	87	47.5
	Unemployed	24	13.1
	Student	42	23.0
	Retired	30	16.4

Self-perceived oral health	Good	32	17.5
	Average	101	55.2
	Poor	50	27.3

Dental experience	Yes	114	62.3
	No	69	37.7

First dental visit	<12 years old	43	23.5
	12–18 years old	82	44.8
	>18 years old	58	31.7

First dental experience	Good	56	30.6
	Not bad	77	42.1
	Bad	50	27.3

Frequency of dental visits	Every 6 months	72	39.3
	Every 12 months	49	26.8
	Less frequent/when needed	62	33.9

Most recent dental experience	Good	108	59.0
	Bad	75	41.0

The length of time since the most recent dental visit	Within 3 months	82	44.8
	3–12 months	54	29.5
	Longer than 12 months	47	25.7

Postponement of the dental visit	Yes	110	60.1
	No	73	39.9

MDAS score		13.63 (3.1)^*∗*^	

MDAS score level	Less	36	19.7
	Moderate	128	69.9
	Severe	19	10.4

^*∗*^Mean (SD), descriptive and frequency analyses.

**Table 2:** Participant responses for the MDAS.

Item name	Not anxious	Slightly anxious	Fairly anxious	Very anxious	Extremely anxious
Before going for treatment	73 (39.9%)	37 (20.2%)	55 (30.1%)	18 (9.8%)	0 (0%)
Waiting for treatment	90 (49.2%)	19 (10.4%)	74 (40.4%)	0 (0%)	0 (0%)
Drilling	19 (10.4%)	36 (19.7%)	18 (9.8%)	110 (60.1%)	0 (0%)
Cleaning	36 (19.7%)	72 (39.3%)	56 (30.6%)	0 (0%)	19 (10.4%)
Local anesthetic injections	37 (20.2%)	0 (0%)	18 (9.8%)	36 (19.7%)	92 (50.3%)

Frequency analysis.

**Table 3:** Association of the MDAS score and other risk factors.

Variables	*X* ^2^ (df)	*p* value
Gender	6.092 (6)	0.413
Age	34.626 (18)	0.011
Educational level	16.742 (18)	0.541
Employment	10.373 (18)	0.919
Self-perceived oral health	3.827 (12)	0.986
Dental experience	1.591 (6)	0.953
First dental visit	25.672 (12)	0.012
First dental experience	0.846 (120)	0.999
Frequency of dental visits	127.511 (12)	0.001
Most recent dental experience	61.196 (6)	0.001
The length of time since the most recent dental visit	110.845 (12)	0.001
Postponement of the dental visit	44.281 (6)	0.001

Chi-square (degree of freedom).

**Table 4:** The correlation between the MDAS score and other variables.

Variables	Spearman correlation	*p* value
Gender	−0.085	0.324
Age	−0.034	0.644
Educational level	−0.096	0.197
Employment	−0.009	0.909
Self-perceived oral health	0.061	0.415
Dental experience	−0.019	0.804
First dental visit	−0.080	0.282
First dental experience	0.010	0.892
Frequency of dental visits	−0.337	0.001
Most recent dental experience	−0.064	0.393
The length of time since the most recent dental visit	−0.062	0.43
Postponement of the dental visit	0.033	0.653

Chi-square (degree of freedom).

## Data Availability

The data used to support the findings of this study are available from the corresponding author upon request.
